# Exercise, Nutrition, and Supplements in the Muscle Carnitine Palmitoyl-Transferase II Deficiency: New Theoretical Bases for Potential Applications

**DOI:** 10.3389/fphys.2021.704290

**Published:** 2021-08-02

**Authors:** Massimo Negro, Giuseppe Cerullo, Mauro Parimbelli, Alberto Ravazzani, Fausto Feletti, Angela Berardinelli, Hellas Cena, Giuseppe D’Antona

**Affiliations:** ^1^Centro di Ricerca Interdipartimentale nelle Attivitá Motorie e Sportive (CRIAMS) – Sport Medicine Centre, University of Pavia, Voghera, Italy; ^2^Department of Movement Sciences and Wellbeing, University of Naples Parthenope, Naples, Italy; ^3^Department of Internal Medicine, University of Pavia, Pavia, Italy; ^4^IRCCS Mondino Foundation, Pavia, Italy; ^5^Department of Public Health, Experimental and Forensic Medicine, University of Pavia, Pavia, Italy; ^6^Clinical Nutrition and Dietetics Service, Unit of Internal Medicine and Endocrinology, ICS Maugeri IRCCS, University of Pavia, Pavia, Italy

**Keywords:** metabolic myopathies, long-chain fatty acids, muscle fatigue, mitochondria, peroxisome, resistance exercise, dietary supplements, muscle pain and rhabdomyolysis

## Abstract

Carnitine palmitoyltransferase II (CPTII) deficiency is the most frequent inherited disorder regarding muscle fatty acid metabolism, resulting in a reduced mitochondrial long-chain fatty acid oxidation during endurance exercise. This condition leads to a clinical syndrome characterized by muscle fatigue and/or muscle pain with a variable annual frequency of severe rhabdomyolytic episodes. While since the CPTII deficiency discovery remarkable scientific advancements have been reached in genetic analysis, pathophysiology and diagnoses, the same cannot be said for the methods of treatments. The current recommendations remain those of following a carbohydrates-rich diet with a limited fats intake and reducing, even excluding, physical activity, without, however, taking into account the long-term consequences of this approach. Suggestions to use carnitine and medium chain triglycerides remain controversial; conversely, other potential dietary supplements able to sustain muscle metabolism and recovery from exercise have never been taken into consideration. The aim of this review is to clarify biochemical mechanisms related to nutrition and physiological aspects of muscle metabolism related to exercise in order to propose new theoretical bases of treatment which, if properly tested and validated by future trials, could be applied to improve the quality of life of these patients.

## Introduction

Despite the advancement of knowledge on the pathophysiology of the carnitine palmitoyltransferase II (CPTII) deficiency, the clinical approach to nutrition and exercise in patients affected by this condition has remained unchanged since its beginning. In fact, since 1973, when the first report of CPTII deficiency was published by DiMauro and DiMauro (1973), patients have been advised to maintain a carbohydrates-rich diet (CHORD), reducing dietary fat, and to restrain from physical activities (rest) to prevent rhabdomyolysis or other unfavorable muscle conditions (Joshi and Zierz, 2020). In contrast to this classic therapeutic protocol (CHORD/rest), only two pioneering studies with promising results are currently available. In the first one, by Roe et al. (2008) seven patients with CPTII deficiency were subjected to a dietary regimen including carbohydrates reduction (37%) in favor to triheptanoin and no episodes of rhabdomyolysis or major muscle complaints were experienced, despite an overall increase in daily physical activity. In the second, more recent study, a case report from our lab (Parimbelli et al., 2021) showed that a high intensity/low duration training program of 6 months was safe and effective in improving body metabolism and aerobic fitness in a 14-year-old CPTII-deficiency girl. From this point of view, it is important to underline that the current recommendations for CPTII deficiency are mainly aimed at reducing the severity of the disease’s symptoms not taking into proper account the long-term consequences of an approach based on CHORD and muscle inactivity on the general health of the subjects. On this topic, current evidence from a large cohort study (Seidelmann et al., 2018) indicated that a high carbohydrates consumption (>70% of energy) was related with a significantly higher risk of all-cause mortality compared with moderate carbohydrates intake (50–55%), and inactivity represents a primary cause that strongly contributes to the onset of several chronic diseases, such as type 2 diabetes, cardiovascular diseases, obesity, cancers, depression, dementia and other neurodegenerative conditions (Booth et al., 2012, 2017; Lavie et al., 2019; Patel et al., 2019). The pathophysiological effects of a lifestyle based on CHORD have been associated with a chronic high glycemic overload that leads to harmful metabolic consequences over time (Augustin et al., 2015; Giugliano et al., 2018). On the other hand, inactivity generates significant adverse organ responses that impair whole-body homeostasis (Schnyder and Handschin, 2015; Rezuş et al., 2020; Romanello and Sandri, 2021).

Considering these important concerns, the aim of this review is firstly to describe how the mitochondrial network and its potential cellular interplay with peroxisomes, which are also involved in the oxidation of fatty acids, may offer new theoretical bases for the management of a CPTII deficiency. Secondly, in order to preserve residual mitochondrial bioenergetics and positively regulate the body homeostasis/adaptation capacities of these patients, the manuscript will put forward an innovative therapeutic view for the management of the disease, based on a diet with a moderate intake of carbohydrates and enriched in proteins, the practice of exercises of suitable type and intensity and a potential use of dietary supplements (Table 1).

**TABLE 1:** Summary of innovative potential treatments for CPTII deficiency patients.

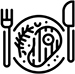	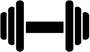	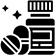
**50–55% Carbohydrates** A higher carbohydrates consumption (> 70% of energy) was related with a significantly higher risk of all-cause mortality	**Exercise suggested** Personalized exercise programs combining HIIT or MIIT (≤70% VO_2_ max) with RT should be encouraged. For beginners or unfit categories, MIIT may be preferred	**Supplements to support muscle bioenergetics, adaptations and functions:** Creatine: 3–5 g/day for 3–4 weeks whey proteins: 20–25 g or EAAs: 10–12 g Vitamin D: 2,000–4,000 IU/day
**25–30% Proteins** High quality protein sources are recommended to optimize muscle recovery and stimulate muscle protein synthesis	**Exercise to avoid** Traditional endurance activities (e.g., running, cycling, swimming) (<65% VO_2_ max) should be avoided in most CPTII deficiency patients	**Supplements to attenuate EIMD and related inflammation:** Omega-3: 1.8–3.0 g/day Beetroot juice: 125–500 mL/day Pomegranate juice: 60–120 mL/day of concentrated product Tart cherries juice: 60 mL/day of concentrated product
**20% Fats** Considered the minimum fats intake to ensure adequacy for essential FAs and fat-soluble vitamins	**Lifestyle to maintain** Physical inactivity should be discouraged	**Supplements for pain management:** Curcumin: 150–2,000 mg/day Ginger: 2,000 mg/day

## The CPTII Deficiency: Update on Pathophysiology and Clinical Background

Among the long-chain fatty acids (LCFAs) utilization diseases, CPTII deficiency is the most common inherited disorder. The clinical importance of this condition is linked to the metabolic role of LCFAs. LCFAs are prevalent substrates in the myocardium at rest and during prolonged exercise in skeletal muscle (Demaugre et al., 1990). LCFAs also provide precursors to build up the membrane lipids and cellular signaling molecules (Stubbs and Smith, 1984; Dutta-Roy et al., 1991; Dutta-Roy, 1994; Uauy et al., 1999). Additionally, LCFAs support the extra energy required in strenuous conditions such as fasting, exposure to cold, fever and emotional stress. As is well known, CPT proteins (CPTI and CPTII) are involved in the transport of LCFAs from the cytosol into the mitochondrial matrix, where they are subsequently oxidized in the form of acyl-CoA esters (Houten et al., 2016).

CPTII deficiency seems to be due to a loss of total enzyme activity and/or an abnormal regulation of the CPTII protein (Joshi and Zierz, 2020). Currently there are three phenotypes of CPTII deficiency described in literature (Joshi and Zierz, 2020): (1) *the lethal neonatal form*, characterized by reduced CPTII enzyme activity in multiple organs, reduced carnitine concentration, and increased concentrations of long-chain acylcarnitine and lipids in serum; (2) *the severe infantile hepato-cardio-muscular form*, characterized by liver failure and cardiomyopathy; (3) *the myopathic form*, characterized by recurrent episodes of muscle pain, weakness, and rhabdomyolysis particularly after long periods of fasting, psychophysical stress or prolonged exercise.

The myopathic form of CPTII deficiency is the most common disorder of lipid metabolism affecting skeletal muscle, frequently associated with myoglobinuria (Wieser et al., 2003). The European prevalence of this condition is estimated at 1–9:100.000 with about 300 cases recognized (Wieser). The defective fatty acids transport into the mitochondria of this form can be highly variable, with a range of residual LCFAs oxidation capacity from 15 to 50% compared to normal subjects (Bonnefont et al., 2004). This may explain, at least in part, why the myopathic form of CPTII deficiency is less severe compared to the other two forms mentioned above, in which the residual capacity to oxidize LCFAs is usually lower. In the myopathic form there are different clinical manifestations among subjects: some affected individuals do not experience muscle weakness between rhabdomyolytic attacks, being asymptomatic for most of time, while others have frequent myalgia even after light to moderate physical activity related to daily life. Despite the myopathic form being sometimes referred to as an ‘adult form’, it can arise from infancy to adulthood (Joshi and Zierz, 2020) and early childhood manifestations have also been reported (Reuschenbach and Zierz, 1988; Bonnefont et al., 1999; Gempel et al., 1999, 2001; Hurvitz et al., 2000; Orngreen et al., 2003; Ørngreen et al., 2005; Anichini et al., 2011; Fanin et al., 2012; Wieser, 2014). Even though there are concerns for the development of kidney disease in CPTII patients due to the high metabolic overload associated to rhabdomyolysis, end-stage chronic renal insufficiency requiring dialysis is only occasionally reported in these patients (Joshi and Zierz, 2020).

### A Difficult Muscle Disease to Diagnose

CPTII muscle deficiency is not easily recognized and patients may have to wait for decades just to get a proper diagnosis, generally based on referred symptoms (Sigauke et al., 2003). Molecular genetic investigation, regarded as the gold standard for the diagnosis, is often performed only after several years of clinical misunderstandings and attempts at treatment. The muscle CPTII disease may be often confused with other, clinically very similar myopathies, such as McArdle disease (MD). MD is an autosomal recessive disorder resulting in myophosphorylase deficiency leading to the inability to use glycogen storages (McArdle, 1951; Llavero et al., 2019). This condition represents the most common cause of acute exercise intolerance in young adults; it is associated with muscle cramps, soreness and recurrent myoglobinuria. However, CPTII deficiency and MD can be functionally differentiated in relation to the onset of some exercise-related signs and symptoms: (1) post-exercise lactate levels are generally higher in CPTII deficiency (>2 mmol/L) (Orngreen et al., 2003) than in MD (<1 mmol/L) (Godfrey et al., 2009); (2) following exercise, muscle disorders appear later in CPTII deficiency and very early in MD (Faigel, 1995); (3) the so-called “second-wind” effect (i.e., an improvement in exercise tolerance with a decrease in perceived exertion and heart rate after 7–8 min of a constant-load exercise protocol) is typically observed in MD patients (Vissing and Haller, 2003; Santalla et al., 2014) and not in CPTII deficiency individuals (Arélin et al., 2020).

An extensive description of the methods for diagnosing CPTII deficiency (i.e., clinical presentation, pathobiochemical characteristics, molecular genetic aspects, detectable biomarkers and genotype-phenotype analysis) has been recently published by Joshi and Zierz (2020). Other possible assessments may consider the electromyography exam, able to demonstrate dysfunction of myoelectric signals related to muscle damage (Sigauke et al., 2003) or functional evaluations, able to show abnormalities in lactate levels, VO_2_ max capacity and respiratory exchange ratio (R) during an ergometric incremental test, which may underline the defective mitochondrial oxidative functionality (Tarnopolsky, 2016).

### CPTII Deficiency and Its Most Dramatic Clinical Syndrome: Rhabdomyolysis

Patients with CPTII deficiency experience recurrent episodes of severe muscle pain associated with rhabdomyolysis (Joshi and Zierz, 2020), in relation to the presence of triggering factors which can produce dramatic and extensive muscle damage due to the altered mitochondrial muscle lipid bioenergetics (Wieser et al., 2003). In particular, these factors include endurance exercise, fasting, exposure to cold and infections, as well as low fluid intake, psychological stress and lack of sleep (Joshi et al., 2019; Joshi and Zierz, 2020). Rhabdomyolysis consists in the partial rupture of myocytes, followed by a dramatic release of several muscle molecules (e.g., creatine kinase (CK), lactate dehydrogenase (LDH), myoglobin (Mb), aminotransferases) in the extra cellular space and then into the blood stream. These molecules are used as blood biomarkers and usually measured when rhabdomyolysis occurs (Bonnefont et al., 1999), even though CK level is considered the most sensitive biomarker (Kim et al., 2016). The normal level of CK is 22–198 U/L. Depending on the degree of rhabdomyolysis, and in relation to the muscle effort sustained and the extension of muscle damage, the level of CK could increase up to 10,000–200,000 U/L observed 24–48 h after the exercise (Criddle, 2003). In addition, muscle pain, electrolyte balance, urine color, arterial blood gas examination, muscle biopsy, and/or electrocardiogram may be used to complete the assessment for the diagnosis of rhabdomyolysis (Kim et al., 2016). The mechanisms involved in the progression of rhabdomyolytic events were elucidated by several authors (Bagley et al., 2007; Kim et al., 2016; Parimbelli et al., 2021). The most severe potential complication of rhabdomyolysis is an acute renal failure due to the dramatic release of Mb by the damaged muscle, resulting in its precipitation and accumulation in renal tubules with an impairment of the filtering efficiency (Panizo et al., 2015).

## Exercise-Therapy in CPTII Deficiency: A Promising Challenge for Future Investigations

### Physical Inactivity, Exercise and Mitochondrial Homeostasis

Physical inactivity is highly harmful and can negatively impair whole-body homeostasis (Schnyder and Handschin, 2015; Booth et al., 2017; Rezuş et al., 2020; Romanello and Sandri, 2021): (1) by increasing chronic systemic inflammation and oxidative stress, which are correlated with the development of many degenerative diseases (e.g., atherosclerosis and osteoarthritis); (2) by decreasing muscle protein synthesis and neuromuscular remodeling, associated with age-related disabling conditions (e.g., sarcopenia and frailty); (3) by compromising the endocrine system and the immune response to infections, thus leading to a decline of the capacity to sustain adequate responses to stress (e.g., injury, acute phases of disease, etc.). Furthermore, muscle disuse leads to a decline of the efficiency of essential processes (i.e., mitochondrial biogenesis, fusion and fission, degradation) necessary for the control of mitochondrial homeostasis (number, morphology and distribution) (Romanello and Sandri, 2021). A loss of mitochondrial homeostasis impairs metabolic adaptation of the mitochondrial network to the cellular bioenergetic requirements and negatively affects skeletal muscle mass, quality and performance, representing a critical factor in the pathophysiology of several muscle-wasting conditions (e.g., aging, intensive care unit-acquired weakness, diabetes, obesity, chronic obstructive pulmonary disease (COPD), cancer cachexia and neuromuscular disorders) (Romanello and Sandri, 2021).

Both inactivity on the one hand and exercise on the other can impact on mitochondrial homeostasis and metabolism very quickly, and this has been recently established comparing the effects of muscle disuse with those of physical rehabilitation on the expression of peroxisome proliferator-activated receptor gamma coactivator-1alpha (PGC-1α) and Sirtuin 3 (SIRT 3) (Buso et al., 2019). PGC-1α is the key factor in the control of mitochondrial biogenesis, morphological and functional integrity, both in physiological conditions and during pathophysiological processes of muscle atrophy and aging (Finck, 2006). SIRT3 is recognized as one of the main mitochondrial activity regulators, with a central role in skeletal muscle (Jing et al., 2011, 2013; Vassilopoulos et al., 2014). In the study by Buso et al. (2019), after 14 days of bed rest a significant decrease in the expression of PGC-1α and SIRT3 was observed, both in the young and the elderly, with an up-regulation of the glycolytic metabolism as a consequence of the mitochondrial impairment. Conversely, in the subsequent 2 weeks of exercise rehabilitation program (3 sessions per week of strength training and high-intensity interval training) the levels of PGC-1α and SIRT3 protein expression showed a remarkable increase in relation to the energy needs, with PGC-1α levels higher than baseline (4.7 times and 2.6 times for young and elderly, respectively) and a shift back to a prevalent mitochondrial oxidative metabolism (Buso et al., 2019). These data are particularly relevant considering that PGC-1α stimulates not only the mitochondrial function but also the peroxisomal biogenesis/activity (Bagattin et al., 2010; Huang et al., 2017), and this effect may positively affect the mitochondria-peroxisome biochemical interplay, a potential alternative pathway to sustaining the energy requirements of exercise in CPTII deficiency patients.

### Mitochondria and Peroxisome Interplay: An Unconsidered Mechanism to Support Energy Production

**FIGURE 1:**
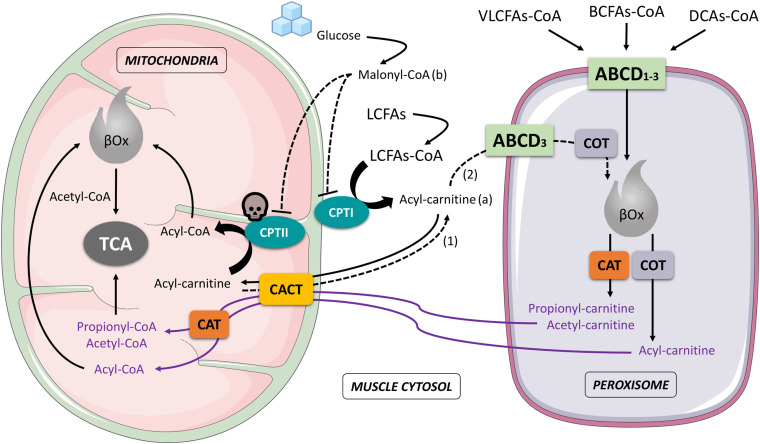
Schematic interaction that involves mitochondria and peroxisome during muscle cell fatty acids oxidation. The figure highlights the importance of this interaction in the case of a CPTII deficiency: Acyl-carnitine accumulated in the cytosol (a), produced by LCFAs-CoA through the activity of CPTI, and coming from the mitochondria (dashed arrow 1), is partially recycled by the peroxisome (dashed arrow 2), subjected to peroxisomal βOx to produce other Acyl-carnitine compounds (e.g., Hexanoyl-carnitine) then reconveyed to mitochondria and reconverted into CoA forms (Acyl-CoA) before being processed in the mitochondrial βOx. In the presence of a high concentration of Malonyl-CoA (b), due to a greater availability of glucose, CPTI and CPTII are both inhibited and LCFAs are mostly deviated to muscle storage (pathways not shown in the figure) instead of being oxidized. ABCD1-3, ATP Binding Cassette subfamily D transporters 1-3; CAT, carnitine acetyltransferase; CACT, carnitine-acylcarnitine translocase; COT, carnitine octanoyltransferase; CPTI, carnitine palmitoyl-transferase I; CPTII, carnitine palmitoyl-transferase II; βOx, beta oxidation; BCFAs-CoA, branched-chain fatty acid-CoA; DCAs-CoA, dicarboxylic acids-CoA; LCFAs-CoA, long-chain fatty acids-CoA; VLCFAs-CoA, very-long-chain fatty acids; TCA, tricarboxylic acid cycle; skull, indicates the CPTII deficiency; arrows and dashed arrows, indicate processes or molecular movements; dashed T-bars, indicate inhibition.

Strong evidence supports a close functional crosstalk between mitochondria and peroxisomes in the process of oxidation of fatty acids (Delille et al., 2010; Dixit et al., 2010; Fransen et al., 2012, 2017; Schrader et al., 2015; Islinger et al., 2018). The β-oxidation (βOx) of fatty acids (FAs) takes place both in mitochondria and peroxisomes, involving, however, different substrates, respectively (Schrader et al., 2015; Wanders et al., 2016): short, medium and long-chain fatty acids (SCFAs, MCFAs and LCFAs) are mainly oxidized in mitochondria (Fransen et al., 2017), whilst very-long-chain fatty acids (VLCFAs), dicarboxylic acids (DCAs) and branched-chain fatty acids (BCFAs) are typically oxidized in peroxisomes (Fransen et al., 2017). Mitochondrial and peroxisomial βOx also lead to the formation of different end-products with relative different metabolic destiny: in the first case, acetyl-CoA is exclusively produced and afterwards directly processed by the tricarboxylic acid cycle (TCA); in the second case, besides acetyl-CoA, other acyl-CoAs (i.e., propionil-CoA, 4,8-dimethylnonanoyl-CoA, adipoyl-CoA, hexanoyl-CoA) are formed, converted in carnitine molecules by carnitine octanoyltransferase (COT) and carnitine acetyltransferase (CAT), then transported across the inner mitochondrial membrane by the carnitine acylcarnitine translocase (CACT) and reconverted in CoAs compounds by a mitochondrial CAT before reaching their final biochemical targets (i.e., mitochondrial βOx or TCA) (Figure 1).

Although less studied and known, the peroxisomial βOx seems to be particularly important considering that its contribution to the total cellular βOx was estimated in mammals from <5% to up to 30% (Thomas et al., 1980; Kondrup and Lazarow, 1985) allowing peroxisomes to perform a pivotal metabolic role in the oxidation of a wide variety of specific FAs. Furthermore, authors suggested that the peroxisome may also be able to oxidize substrates that are typically handled by mitochondria (Vamecq and Draye, 1989; Vamecq et al., 1989; Ferdinandusse et al., 2004), especially when specific mitochondrial pathways are inhibited (Violante et al., 2013, 2019). On this topic remarkable studies have been carried out by Violante et al. (2013, 2019), which demonstrated, *in vitro* and *in vivo*, that peroxisomes can accept and oxidize MCFAs and LCFAs, typically oxidized in mitochondria, becoming metabolically relevant in presence of mitochondrial fatty acid oxidation disorders, including CPTII deficiency.

An extensive description of the metabolic interactions between mitochondria and peroxisomes in the management of fatty acid oxidation has recently been published by Houten et al. (2020). Although the magnitude of mitochondria-peroxisomes interaction in the CPTII deficiency is yet to be established in humans, we can speculate that this “unconsidered” cooperative role of peroxisomal βOx may represent a key process and be potentially trainable by an appropriate program of physical exercise.

### No Rest: The Role of Intermittent Training and Resistance Exercise in CPTII Deficiency

Since patients carrying the myopathic form of CPTII deficiency display normal fat turnover at rest (Orngreen et al., 2002) and physical exercise was recognized as the most common trigger factor involved in myalgia and/or rhabdomyolytic events, current recommendations exclude the practice of all types of physical activities, in particular prolonged and strenuous exercise, and complete rest is still recommended (Joshi and Zierz, 2020). However, this approach does not properly take into account the variability of metabolic requests that occur in relation to different exercise intensities and/or durations, and how these metabolic requests can affect macronutrient utilization. Indeed, during submaximal exercise intensities (<65% VO_2_ max), especially if maintained beyond 90 min (Purdom et al., 2018), FAs are the prevalent fuel source of muscle energy, with a maximal FAs utilization (maximal fat oxidation, MFO) between 45 and 65% of VO_2_ max. At these intensities, as in endurance activities, the contribution of carbohydrates oxidation is relatively low. On the other hand, for exercise intensities that exceed MFO, with duration typically lower than 90 min (as in intermittent training, stop-and-go activities and/or resistance exercise), the utilization of carbohydrates is predominant, mainly as a muscle glycogen (Purdom et al., 2018), phosphocreatine (PC) resynthesis is involved for a rapid ATP regeneration (Hargreaves and Spriet, 2020), and the use of FAs to maintain energy availability is sharply reduced (Purdom et al., 2018). Considering this physiological background, traditional endurance activities (e.g., running, cycling, swimming, etc.) should certainly be avoided in most CPTII deficiency patients, especially if unconditioned to exercise, as they are unable to properly satisfy the increase of muscle energy demand from FAs oxidation. Conversely, high intensity interval training (HIIT) or resistance training (RT) can be safely performed in CPTII disorders because the ATP turnover of these activities is mainly based on PC/glycogen pathways, which are totally preserved and optimizable in these patients.

HIIT is commonly defined as relatively intense bouts of exercise that elicit ≥80% of maximal heart rate (MHR), interspersed by periods of lower intensity exercise or rest for recovery (Weston et al., 2014). HIIT has received considerable attention in recent years for its metabolic effects, which are higher than in aerobic continuous training, with clinical relevance both in healthy and diseased subjects (Alkahtani et al., 2013; Jiménez-Pavón and Lavie, 2017; Gibala, 2018). The capacity of interval training to elicit greater health benefits than continuous exercise, matched for duration and intensity, seems to be related to the intrinsic nature of the intermittent mechanical work (Jiménez-Pavón and Lavie, 2017; Gibala, 2018). Notably, on this topic authors suggest that some cellular signaling pathways that regulate skeletal muscle mitochondrial biogenesis and function (i.e., AMPK, p38-MAPK, CaMKII and PGC-1α) are activated to a greater extent after intermittent activity compared with continuous exercise, even when relative intensity is moderate (Combes et al., 2015). However, since HIIT exercise mode can discourage physically inactive individuals and/or can be inappropriate in certain conditions (e.g., untrained obese), a moderate-intensity intermittent training (MIIT) may be preferred. Furthermore, MIIT does not require supervision and can generally be easier to put into practice for unfit categories (Jiménez-Pavón and Lavie, 2017). Comparative studies have shown that HIIT and MIIT have similar effects on several metabolic outcomes, such as fats oxidation (Alkahtani et al., 2013), vascular function (Rakobowchuk et al., 2012), body composition, insulin resistance and low-density lipoprotein cholesterol (LDL) (Racil et al., 2013).

Based on the specific involvement of type II muscle fibers, RT plays a key role in the maintenance of adequate muscle mass, strength and power, which are fundamental to supporting movement control, walking speed and functional independence (Westcott, 2012). RT has a strong clinical background for the prevention and management of type 2 diabetes, improving visceral fat quantity, HbA1c blood levels, glucose transporter type 4 distribution, and insulin sensitivity (Westcott, 2012; Codella et al., 2018; Mcleod et al., 2019). RT also produces cardiovascular benefits, by reducing resting blood pressure, LDL and triglycerides, and increasing high-density lipoprotein cholesterol (HDL). Furthermore, RT may promote bone development and prevent muscle loss due to aging, decreasing pain sensibility typically associated with arthritis or other inflammatory diseases that can even affect CPTII deficiency patients, especially during the advanced stage of life. Less clear are the effects of RT on mitochondrial biogenesis or oxidative capacity, with mixed results (Parry et al., 2020). However, given that RT may increase VO_2_ max in untrained individuals, it is plausible that some aspects of the mitochondrial function may be enhanced with this paradigm of exercise (Parry et al., 2020), even though this assertion is only speculative at this stage and more investigation should be run in this area. No information is available on the effect of HIIT/MIIT or RT and peroxisomal biogenesis/oxidative capacity or mitochondria-peroxisomes interaction.

The first trial that successfully applied MIIT and RT on CPTII deficiency patients has recently been reported by our lab (Parimbelli et al., 2021), with promising results. In the study, a 14-year-old female with CPTII deficiency was involved in an exercise program of 6-months (3-days per week, 1 h/session each) based on MIIT at 70% VO_2_ max and resistance upper/lower split workouts. After 6 months, indirect calorimetry revealed improved resting metabolic rate (RMR) and reduced respiratory quotient (RQ). Furthermore, the cardiopulmonary exercise test (CPET) revealed remarkable increases in peak power output and isocapnic buffering period. Importantly, this study also showed that the training program was absolutely safe and sustainable, with no significant post-exercise changes in blood CK, and no muscle pain and/or rhabdomyolysis attacks were experienced by the patient during the experimental protocol. Improvement of cardiovascular and muscular adaptations (including mitochondrial content and biogenesis) were only speculated by the authors at this stage of knowledge, and this requires further examination.

## Nutritional Therapy in CPTII Deficiency: Is the High Carbohydrates Diet the Right Way?

The current nutritional approach for CPTII deficiency generally consists of a CHORD (at least 65-70% of energy), with restricted fats (no more than 20%) and a daily proteins intake of about 15%. This nutrition strategy was followed for 3 days by four patients before an exercise test (cycle ergometer at 50% of individual VO_2_ max until exhaustion) and turned out to be more effective in reducing the rate of perceived exertion and increasing muscle work duration compared to a control diet (60% fats, 15% proteins, and 25% carbohydrates) (Orngreen et al., 2003). Although the CHORD can increase the short-term tolerance to sub-maximal exercise intensity (Orngreen et al., 2003), it does not seem to prevent recurrent muscle weakness, myalgia and rhabdomyolysis attacks (Roe et al., 2008; Schnedl et al., 2020). Furthermore, a CHORD could also be inadequate for a long-term overall health management of these patients, considering the possible negative effects on glucose homeostasis and fat metabolism over time (Phillips et al., 2020).

As authors recently underlined, a chronic exposure to CHORD promotes hyperglycemic stimulus with hyperinsulinemic response (Phillips et al., 2020) and this may reduce muscle fatty acid oxidation in humans (Rasmussen et al., 2002). Hyperglycemia with hyperinsulinemia functionally reduces the CPTI activity in muscle cell, involving the regulatory role of malonyl-CoA which increases following a greater availability of glucose (Rasmussen et al., 2002; Figure 1). In addition, in CPTII deficiency, unlike the physiological condition which CPTII enzyme is insensitive to changes in malonyl-CoA concentration (Murthy and Pande, 1987; Woeltje et al., 1990), the activity of CPTII can also be inhibited by malonyl-CoA (Motlagh et al., 2016). The inhibitory effect is more pronounced in the variant S113L than in the wild type of the disease, confirmed by *in-vitro* results that clearly showed an enzymatic residual activity of about 40 and 70%, respectively, after pre-incubation with malonyl-CoA (Motlagh et al., 2016). The inhibition of CPTI and CPTII by malonyl-CoA, taken together, may produce a significant decrease in the transport of LCFAs from cytosol into the mitochondria, diverting the LCFAs metabolic fate toward storage in the muscle as triglycerides (Rasmussen et al., 2002). The final clinical impact of the process described is related to the fact that elevated fat stores within the muscle cell play a role in the development of insulin resistance (Storlien et al., 1991; McGarry, 1992; Pan et al., 1997), with even more dramatic consequences on glucose and fats metabolism when a CHORD is combined with a lifestyle based on inactivity, as currently suggested in CPTII deficiency.

Based on the above it should be necessary to consider that for most CPTII deficiency patients the current guidelines on carbohydrates intake may not be adequate for the prevention of muscle conditions due to the disease, or for satisfying the metabolic requests of fat oxidation, potentially decreasing the already compromised mitochondrial transport capacity. Other possible nutrient ratios need to be investigated, especially as regards carbohydrates Vs proteins (e.g., 50–55% carbohydrates and 25–30% proteins) as described by Scott et al. (1991), maintaining fats intake at 20% of energy (considered the minimum fats intake to ensure adequacy for essential FAs and fat-soluble vitamins) (Harcombe, 2019). Reducing the carbohydrates content of the diet, by replacing a part of carbohydrates with proteins, may have several positive effects: (1) decreasing both hyperglycemia and hyperinsulinemia, due to different glycemic regulation of proteins compared to carbohydrates (Phillips et al., 2020); (2) increasing fatty acid oxidation in skeletal muscle, reducing muscle fat deposition and preventing insulin resistance (Phillips et al., 2020); (3) stimulating muscle protein synthesis (Phillips et al., 2020) to support muscle recovery after MIIT and RT; (4) generating a part of glucose, *via* gluconeogenesis, but in a slower way than directly from the carbohydrates (Phillips et al., 2020). Recommendations to use carbohydrates-concentrated foods, snacks, sports bars or liquid beverages should remain limited to MIIT and/or RT sessions (before, during and soon after), with the aim of supporting exercise time-related energy delivery (Parimbelli et al., 2021).

## Potential Supplements in CPTII Deficiency

Apart from some attempts based on the use of fibrates (Djouadi and Bastin, 2008; Bonnefont et al., 2010), at present there are no approved drugs for the treatment of the disease. This has led researchers to speculate on possible adjuvant approaches based on the use of dietary supplements. Some compounds have historically been suggested to support lipid metabolism (i.e., carnitine, medium chain triglycerides), with the aim of positively impacting on the frequency of muscle events, although the literature shows concerns and/or conflicting positions on this matter (Roe et al., 2008; Gnoni et al., 2020; Sawicka et al., 2020). Other supplement categories (e.g., creatine, essential amino acids, etc.) with strong evidence in muscle health/performance, but not conventionally used in CPTII deficiency and only speculative at this stage, could instead be potentially proposed to promote muscle bioenergetics and remodeling and/or to manage clinical symptoms in these patients (Table 1).

### Supplements to Support Muscle Lipid Metabolism (Carnitine, Medium Chain Triglycerides)

Based on a biochemical background, the use of carnitine has been speculated for CPTII deficiency by several authors in the past, even though this practice has never been investigated in these patients. However, considering that to date no scientific basis supports significant improvement of muscle carnitine content and/or performance in healthy subjects or athletes after a long lasting supplementation (Gnoni et al., 2020) and that carnitine can be partly degraded to trimethylamine-N-oxide (TMAO), which is considered a novel risk factor for cardiovascular diseases (Arsenian, 1997; Randrianarisoa et al., 2016; Tang and Hazen, 2017; Ding et al., 2018; Gao et al., 2019), a chronic supplementation of carnitine should be discouraged in these patients. In addition, in muscle CPTII deficiency acyl-carnitine is only partially transported across the inner mitochondrial membrane and the conversion of acylcarnitine into acyl-CoA is anyway insufficient to justify carnitine supplementation (Gnoni et al., 2020; Figure 1).

Medium chain triglycerides (MCTs) are able to cross the mitochondrial membranes independently of CPT transporters and CAT (Jeukendrup et al., 1998), and this has led to speculation on a possible use of MCTs in CPTII deficiency. Despite this background, conflicting opinions emerge on this topic, with some authors suggesting their use to sustain muscle bioenergetics and reduce plasma triglycerides (Scott et al., 1991; Bonnefont, 2004; Deschauer et al., 2005), while others do not consider MCTs effective in controlling muscle pain, avoiding rhabdomyolysis, or improving exercise performance and muscle recovery (Carroll et al., 1978; Roe et al., 2008). An interesting contribution on MCTs comes more recently from Violante et al. who, by using CPTII- and CACT-deficient cell lines, demonstrated how the majority of MCTs require carnitine shuttles to access the mitochondrial matrix and only 25% may cross the mitochondrial membranes by diffusion, underlining that when the CPTII is defective MCTs can be partially oxidized in peroxisomes (Violante et al., 2013). However, currently we don’t know how the use of MCTs may modulate the mitochondria-peroxisome interplay during different conditions of diet and exercise, and how this may regulate muscle metabolism in CPTII deficiency patients. At present, therefore, the potential use of MCTs to counter oxidative requests and prevent muscle symptoms in these subjects needs further evaluation.

### Supplements to Support Muscle Bioenergetics, Adaptations and Functions (Creatine, Proteins/EAAs, Vitamin D)

Creatine is one of the most popular nutritional ergogenic aids for athletes (Kreider et al., 2017). Studies have consistently shown that creatine supplementation can improve exercise performance, through an increase of intramuscular PC concentration, and may enhance training adaptation/muscle hypertrophy by controlling molecular pathways (e.g., MRF-4, Myf-5, Myo-D, and myogenin) involved in muscle protein expression (Negro et al., 2019). Furthermore, creatine supplementation might influence post-exercise muscle recovery by enhancing muscle satellite cell proliferation (Vierck et al., 2003; Olsen et al., 2006). Creatine monohydrate (CM) is currently considered a safe and effective supplement to support resistance activities in the healthy, and several therapeutic benefits in diseased populations (ranging from the young to the elderly) have been reported (Smith et al., 2014; Candow et al., 2019; Stares and Bains, 2020; Jagim and Kerksick, 2021; Kreider and Stout, 2021). Considering its strong background, a periodical use of CM can be speculated in CPTII deficiency patients with different aims (e.g., at the beginning of a training program, to support muscle bioenergetics or to reduce fatigue when muscle recovery/adaptation needs to be boosted).

An important body of evidence suggests that a higher amount of proteins is required by active individuals to optimize exercise training adaptations and muscle remodeling (Cermak et al., 2012; Devries and Phillips, 2015; Churchward-Venne et al., 2016; Jäger et al., 2017; Morton et al., 2018). Dietary proteins and/or essential amino acids (EAAs), positively acting on the balance between protein synthesis and breakdown, can increase muscle mass or attenuate muscle loss, even during immobilization and aging (Cholewa et al., 2017). Furthermore, considering the capacity of EAAs to regulate ATP production/utilization (Dioguardi, 2004; Pasini et al., 2004) a possible role of these compounds in energy delivery during fatiguing tasks has recently been speculated by our lab (Negro et al., 2018). Based on the above, to satisfy the daily proteins intake to partly sustain the energy cost of strength activities and to promote muscle recovery after exercise sessions, a periodical supplementation with protein powders (e.g., whey proteins) or EAAs may be suggested in these subjects. This recommendation could be even more important in the presence of structural alteration of fibers as mirrored by increased blood CK levels, to prevent possible progression to rhabdomyolysis.

There is a strong association between the nutritional status of vitamin D and an optimal muscle function (Bhattoa et al., 2017). Indeed, through its binding to muscle vitamin D receptors, vitamin D mediates genomic and non-genomic effects in muscle cells, promoting muscle contractility through calcium uptake, myoblast differentiation, and the insulin sensitivity (Dirks-Naylor and Lennon-Edwards, 2011; Christakos et al., 2016; Bhattoa et al., 2017). Large cross-sectional studies underline a relationship between an insufficient level of serum 25(OH)D (<50 nmol/L) and low physical performance (Bischoff-Ferrari et al., 2004; Houston et al., 2007, 2011, 2012; Dirks-Naylor and Lennon-Edwards, 2011; Toffanello et al., 2012), mobility (Bischoff-Ferrari et al., 2004; Houston et al., 2011, 2012; Toffanello et al., 2012), muscle strength (Mowé et al., 1999; Zamboni et al., 2002; Houston et al., 2007, 2011, 2012; Toffanello et al., 2012), and greater disability (Zamboni et al., 2002; Houston et al., 2011). The importance of considering baseline serum 25(OH)D concentrations has been emphasized, since individuals with vitamin D deficiency appear to be more responsive to supplementation (Annweiler et al., 2009). Considering these findings, CPTII deficiency patients need to be screened to evaluate vitamin D blood levels, and in relation to the concentration detected a dietary supplementation should be suggested.

### Supplements to Attenuate Exercise-Induced Muscle Damage and Related Inflammation (Omega3, Polyphenols)

Exercise-induced muscle damage (EIMD) is a physiological regulated process of degradation and repairing, closely related to muscle adaptation and remodeling, characterized by muscle soreness and transient lower muscle function, in which several cell types are involved (i.e., neutrophils, macrophages, lymphocytes) along with an increase of reactive oxygen spices (ROS), the serum rise of various inflammatory cytokines (e.g., IL-1β, IL-6), and a release of intramuscular proteins (e.g., CK, LDH) due to damage (Byrne et al., 2001; Marcora and Bosio, 2007). EIMD, within certain limits, must also take place in CPTII deficiency subjects and is essential for their muscle recovery and adaptation to mechanical work. However, since the muscle bioenergetics is compromised in these patients, their degree of EIMD can be greater than in normal subjects, with higher levels of oxidative stress and inflammation, and this also results in a longer muscle repair time. To reduce the impact of EIMD and to favor faster muscle recovery, the use of anti-inflammatory supplements, such as omega-3 polyunsaturated fatty acids (PUFA), and/or functional foods rich in antioxidants (e.g., juices rich in polyphenols) can be beneficial in these patients. PUFA have a well-established capacity to reduce markers of EIMD (CK, LDH, IL-1β, IL-6) measured after high-damaging protocols (eccentric exercise), and after different duration times of supplementation, from 30 days (Tartibian et al., 2011) to 10 weeks (Ramos-Campo et al., 2020). Concentrated juices (e.g., red beetroot, pomegranate and tart “Montmorency” cherries) can exert positive effects on the magnitude of EIMD and its resolution (Owens et al., 2019; Bongiovanni et al., 2020) through several mechanisms that involve polyphenols effects: (1) by lowering free radical production and lipid peroxidation (Ammar et al., 2018); (2) by reducing inflammation transcripts and molecules related to perceived soreness after damage (e.g., NF-κB, TNF-α and cyclo-oxygenase-2 (COX-2) (Ammar et al., 2018; Kashi et al., 2019); (3) by improving muscle perfusion and oxygen extraction due to their vascular function (Kashi et al., 2019).

### Supplements for Pain Management (Curcumin, Ginger)

Single or multiple attacks of mild to severe myalgia, even not exercise-induced, are common symptoms in CPTII deficiency, with at least 50 attacks per year (Joshi et al., 2019; Joshi and Zierz, 2020). Based on this, pain management is another important clinical target in these patients, with the aim to improve the quality of their life. Nutritional compounds, such as curcumin and ginger, may improve pain symptoms through several mechanisms (Perna et al., 2020) and, therefore, they may be potentially used in these patients if necessary. Curcumin has a significant effect in reducing the production of recognized algogenic substances, such as COX-2 molecules (Kang et al., 2004), prostaglandin E2 (Clutterbuck et al., 2013) and histamine (Nugroho et al., 2009) and can decrease pain sensitivity both by interacting with nociceptor response (Yeon et al., 2010; Leamy et al., 2011), and by modulating pro-inflammatory cytokines production, such as TNF-α, IL-6, and IL-8 (Fernández-Lázaro et al., 2020). Likewise, ginger can exhibit pain-reducing effects by interacting with COX-2 pathways, by inhibiting inflammatory cytokines transcripts (*via* NF-κB), and by acting as an agonist of vanilloid nociceptors (Rondanelli et al., 2020).

## Conclusion

An impaired fats oxidation capacity in skeletal muscles defines CPTII myopathy as a useful model of metabolic fatigue in which intolerance to endurance exercise and the onset of muscle symptoms are strictly interconnected. However, considering that physical inactivity results in fat gain and muscle loss over time, with dramatic metabolic consequences perhaps even more serious than the disease itself, forms of physical exercise other than endurance (i.e., intermittent and/or resistance) should be necessarily considered in these patients to safely promote health, fitness, and quality of life. Although a positive impact of chronic metabolic adaptations induced by MIIT and/or RT on muscle endurance capacity cannot be excluded in these subjects, how these forms of exercise may improve the residual mitochondrial functions and potentially promote the mitochondria-peroxisomes interactions in a CPTII disorder remains to be clarified. Future research efforts will certainly have to focus on these topics, considering age, gender, different carbohydrates/proteins intake and the metabolic responses to the evidence-based supplements discussed.

## Author Contributions

All authors contributed to the article and approved the submitted version. MN and GD’A conceived the original idea of the manuscript. MN and GC contributed equally to this work as main authors. AB, HC, and GD’A revised the manuscript before submission.

## Conflict of Interest

The authors declare that the research was conducted in the absence of any commercial or financial relationships that could be construed as a potential conflict of interest.

## Publisher’s Note

All claims expressed in this article are solely those of the authors and do not necessarily represent those of their affiliated organizations, or those of the publisher, the editors and the reviewers. Any product that may be evaluated in this article, or claim that may be made by its manufacturer, is not guaranteed or endorsed by the publisher.
